# Prognostic impact of non-improvement of global longitudinal strain in patients with revascularized acute myocardial infarction

**DOI:** 10.1007/s10554-021-02349-2

**Published:** 2021-07-29

**Authors:** Jan Erik Otterstad, Ingvild Billehaug Norum, Vidar Ruddox, An Chau Maria Le, Bjørn Bendz, John Munkhaugen, Ole Klungsøyr, Thor Edvardsen

**Affiliations:** 1 Department of Cardiology, Vestfold Hospital Trust, Tønsberg, Norway, P. O Box 2168, 3103 Tønsberg, Norway; 2 Faculty of Medicine, University of Oslo, Oslo, Norway, Blindern, P.O Box 1078, 0316 Oslo, Norway; 3 Division Rikshospitalet, Department of Cardiology, Oslo University Hospital, Oslo, Norway, Nydalen, P.O Box 4950, 0424 Oslo, Norway; 4 Department of Medicine, Drammen Hospital Trust, Drammen, Norway, Vestre Viken HF, P.O box 800, 3004 Drammen, Norway; 5 Oslo Centre for Biostatistics and Epidemiology, University of Oslo, Oslo, Norway, Sogn Arena 3.etg, Nydalen, P.O Box 4950, 0424 Oslo, Norway

**Keywords:** Global longitudinal strain improvement; PCI treated myocardial infarction, Prognostic impact

## Abstract

Global longitudinal strain (GLS) is a more sensitive prognostic factor than left ventricular ejection fraction (LVEF) in various cardiac diseases. Little is known about the clinical impact of GLS changes after acute myocardial infarction (AMI). The present study aimed to explore if non-improvement of GLS after 3 months was associated with higher risk of subsequent composite cardiovascular events (CCVE). Patients with AMI were consecutively included at a secondary care center in Norway between April 2016 and July 2018 within 4 days following percutaneous coronary intervention. Echocardiography was performed at baseline and after 3 months. Patients were categorized with non-improvement (0 to − 100%) or improvement (0 to 100%) in GLS relative to the baseline value. Among 214 patients with mean age 65 (± 10) years and mean LVEF 50% (± 8) at baseline, 50 (23%) had non-improvement (GLS: − 16.0% (± 3.7) to − 14.2% (± 3.6)) and 164 (77%) had improvement (GLS: − 14.0% (± 3.0) to − 16.9% (± 3.0%)). During a mean follow-up of 3.3 years (95% CI 3.2 to 3.4) 77 CCVE occurred in 52 patients. In adjusted Cox regression analyses, baseline GLS was associated with all recurrent CCVE (HR 1.1, 95% CI 1.0 to 1.2, p < 0.001) whereas non-improvement versus improvement over 3 months follow-up was not. Baseline GLS was significantly associated with the number of CCVE in revascularized AMI patients whereas non-improvement of GLS after 3 months was not. Further large-scale studies are needed before repeated GLS measurements may be recommended in clinical practice.

*Trial registration*: Current Research information system in Norway (CRISTIN). Id: 506563

**Supplementary Information:**

The online version contains supplementary material available at 10.1007/s10554-021-02349-2.

## Introduction

The majority of patients who have been treated with early percutaneous coronary intervention (PCI) for acute myocardial infarction (AMI) are discharged without reduced left ventricular ejection fraction (LVEF) [1–3]. Reduced global longitudinal strain (GLS) is a more sensitive predictor for cardiac events and remodeling than LVEF post-AMI, especially among patients without reduced LVEF [3–6]. The question arises whether improvement of GLS following AMI is associated with favorable clinical outcomes, such as recently reported for improvement of LVEF [7]. In a study of patients treated with PCI for ST-segment elevation myocardial infarction (STEMI), Antoni et al. [8] reported significant improvement of GLS at 3 months and between 3 and 12 months. Left anterior descending artery as culprit vessel, peak cardiac troponin T level and diastolic function were the independent predictors for improvement. Baron et al. [9] studied post AMI patients of whom 47% had STEMI and 90% had been treated with PCI. GLS significantly improved during the following 12 months, with independent predictors being initial impairment of LV function, including assessment with GLS, male gender, non-smoking and treatment with beta-blockers. None of these studies, however, explored the association between the improvement or non-improvement of GLS and subsequent composite cardiovascular events (CCVE).

In the present study, we aimed to explore the association between GLS improvement or non-improvement at baseline and 3 months following PCI treated AMI and clinical outcome during more than 3 years follow-up. Our hypothesis was that non-improvement of GLS predicted a higher incidence of CCVE than improvement.

## Methods

### Design and study population

A prospective, observational follow-up study of patients recruited from Vestfold Hospital Trust, a non-invasive, secondary care general hospital. Since 2005, all eligible patients with AMI have been transferred for invasive revascularization therapy at Oslo University Hospital (tertiary center). Following PCI, most patients return within 1–2 days for subsequent management at Vestfold. Stabilized patients were included consecutively within 4 days following PCI from April 1st 2016–July 31nd 2018 according to the following criteria:

- The diagnosis of AMI type 1 [10] and PCI performed according to prevailing guidelines [11] at the time when the study was planned (2015).
- Providing written consent for study participation.

### Exclusion criteria

#### Baseline

- Hemodynamically unstable patients (ongoing arrhythmia, heart failure, significant comorbidities) according to the opinion of the principal investigators.
- Atrial fibrillation / flutter / irregular heart rhythm
- Poor echocardiographic images according to the principal investigator`s opinion.

#### 3 months

- Not willing to attend the second examination
- Recurrent AMI, development of heart failure requiring hospitalization or arrhythmias between the two echocardiographic studies.
- Anticipated inability to attend the 3 months examination and life expectancy < 2 years

The study was approved by the Regional Ethics Committee of Health Region South-East, Norway (2015/2359).

### Echocardiography

All echocardiographic examinations were performed with a General Electric scanner Vivid E9 (Vingmed Ultrasound, Horten, Norway) within 4 days of intervention. Whenever feasible, a new echocardiographic examination was performed after 3 months. In order to optimize the quality of recordings and minimize the influence of interobserver variability only two experienced operators (VR and JEO) performed the echocardiographic examinations.

### Global longitudinal strain

Three to four consecutive heartbeats were recorded from each of the three apical views. End of systole was defined as aortic valve closure registered by continuous Doppler. Manual editing of the region of interest was performed, whenever found appropriate by the investigator, and in accordance with the recent recommendations [12]. As part of quality insurance all baseline and 3 months GLS values were reanalyzed blindly, and in case of a deviation of > 10%, a reassessment was performed of the registered value based upon consensus between the two echocardiographers involved in the study. We excluded images with suboptimal tracking of the endocardium in more than one segment in one single view, or if frame rate was below 50 Hz.

Improvement was defined as a relative decrease of GLS (i.e. to a more negative value) in percentage of the baseline value from ÷ 0% to ÷ 100%; and non-improvement as a relative increase (i.e. to a less negative value) from 0 to 100%. Patients with identical GLS on both occasions were categorized as improvers.

### Conventional echocardiography

LV mass index was evaluated by M-mode in parasternal long axis. LVEF was measured by the biplane Simpson method. Maximal left atrial volume index (LAVI) was measured by the biplane area-length method. Pulsed and tissue Doppler were applied for E/e’ ratio using the average of e’ septal and e’ lateral velocities.

### Follow up

Patients were followed until death or 31st October 2020 by telephone interviews and careful screening of all available hospital records. All patients were offered participation in our multidisciplinary cardiac rehabilitation program [13] and medical treatment was given according to present guidelines. The following CCVE were defined from the 3 months follow-up echocardiogram to the end-of follow-up, as judged from all available hospital records by two independent investigators who were blinded to the echocardiographic findings:

1. Death
2. Reinfarctions according to the universal definition [10]
3. Hospitalization for heart failure
4. Hospitalization for angina pectoris with a new coronary angiogram confirming progression of coronary artery stenoses requiring urgent PCI, or if not due to poor periphery of stenotic coronary arteries
5. Hospitalization for ventricular arrhythmia
6. New diagnosis of atrial fibrillation (AF) during follow-up if it was documented in a 12-channel ECG provided not being present before the index AMI
7. Hospitalization for stroke / transitory ischemic attack

### Statistical analysis

Data is either presented as mean ± SD or median and interquartile range (IQR) as appropriate. Normality was tested with the Shapiro–Wilk test, whenever visual assessment of the distribution was dubious. Differences of variables at baseline between the two groups according to change in GLS (from baseline to 3 months) were assessed with analysis of variance and Kruskal–Wallis for normal and non-normal distributions respectively. A chi square test or Fisher’s exact test was used for differences in categorical parameters. Survival analysis was performed to compare time from baseline to CCVE in the two groups, and identify covariates associated with the length of this time-interval. The time-scale was observation time, from operation to CCVE or stop of follow-up, which ever came first. Right censoring at end of follow-up occurred for those without CCVE. A Kaplan–Meier plot was used to compare time to CCVE between the two groups visually with corresponding log-rank test. Cox proportional hazards regression was performed to estimate relative incidence rates (hazard ratios) of CCVE and adjusted for relevant baseline covariates. In addition to assess the association between improvement of GLS and clinical outcome we introduced subsequent events as outcome in order to obtain higher precision. Tests for the assumption of proportional hazards were performed. We chose 2 models, testing both the hazard rates for first and for all (first + subsequent events) CCVE.

Model 1: A simplified approach including age decades, gender, baseline GLS and the two delta GLS categories using patients with non-improvement as reference (HR = 1).

Model 2: An extension with addition of the following co—variables (baseline): Previous AMI, STEMI with anterior wall location and baseline LVEF, also with the non-improvement group as reference.

For those with subsequent events, a sandwich variance estimator was used to account for dependence. The “survival” R-package was used for analysis [14]. All other statistical analysis were performed using SPSS version 25 (SPSS, Inc, Chicago, IL).

### Reproducibility of repeated GLS measurements

Intra- and interobserver variability of GLS in our laboratory was tested in a previous study in 20 randomly selected patients. For the interobserver analyses, two observers investigated the same cine-loops blinded to the results of the other. For intra-observer analyses, one observer investigated the same cine-loops approximately 4 weeks apart. Intraclass correlation coefficient was 0.91 (0.77–0.97) for intraobserver variability and 0.84 (0.57–0.94) for interobserver variability.

## Results

**Fig. 1:**
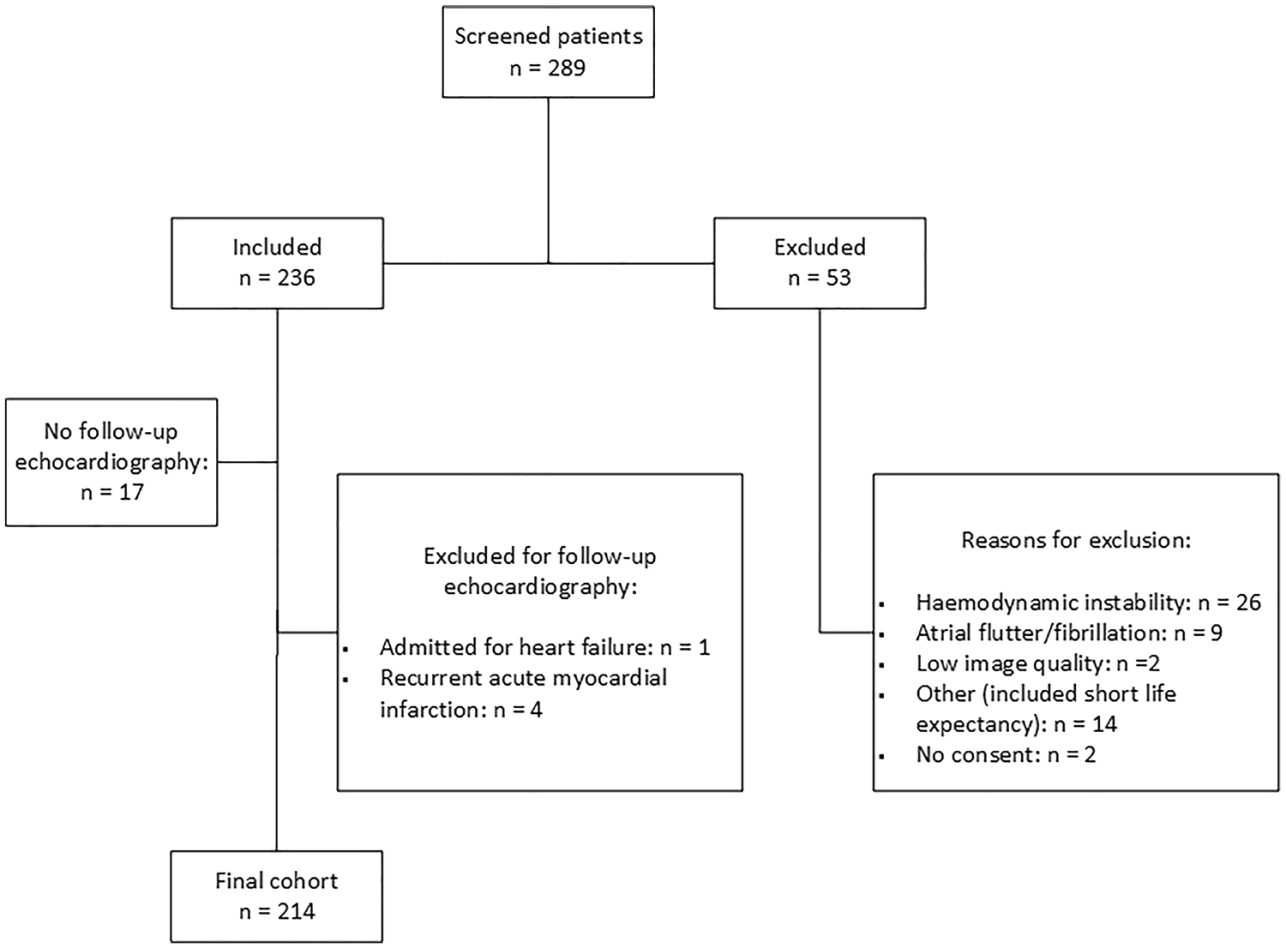
Flow chart of patients screened and included in the present study

In all, 289 patients were screened for participation and reasons for exclusion in 53 patients are provided in Fig. 1. Of the remaining 236 patients, one patient (0.4%) was excluded due to hospitalization for heart failure, four (1.7%) due to recurrent AMI during the first 3 months and 17 (7.2%) did not wish to attend. Thus, 214 patients were eligible for the final study. There were no significant differences in clinical and echocardiographic characteristics between the total population included (n = 236) and the final study group (n = 214) at baseline (Supplemental file 1 and 2). Mean age in the study group was 65 (± 10) years, 25% were females, mean LVEF was 50% (± 8), 22 patients (10.3%) had LVEF < 40% and 17% had a previous MI. The percentage of STEMI was 53%, all treated with primary PCI at a median of 4.0 (IQR 3.0) hours after debut of symptoms. NSTEMI patients were treated with PCI after a median of 48 (IQR 37) hours. All patients were examined with baseline echocardiography after a median of 2 (IQR 1) days following PCI.

After 3 months follow-up, mean GLS had improved from − 14.4% (± 3.4) to − 16.2% (± 3.4), p < 0.001) i.e., a relative mean improvement of 12.5% of the baseline value. At that time GLS was not improved in 50 (23%) and improved in 164 patients (77%), of whom five had identical GLS on both occasions. Baseline characteristics are provided in Table 1. Patients with non-improvement had a higher number of stents implanted (p < 0.01) than improvers. In the subgroups with STEMI those with non-improvement had a longer mean symptom to revascularization time than improvers (p < 0.001). There were no significant difference between the number of patients where time to primary PCI was ≥ 10 h between improvers and non-improvers presenting with STEMI (p = 0.53).

**Table 1:** Clinical characteristics at baseline and patient management plan at discharge and 3 months

	Improvement (n = 164)	Non-improvement (n = 50)
Clinical characteristics		
Age	65 (10)	65 (10)
Female, n (%)	37 (23)	15 (30)
Heart rate, (bpm)	70 (12)	71 (11)
Systolic blood pressure (mmHg)	127 (18)	128 (18)
Previous MI, n (%)	26 (16)	10 (20)
Diabetes mellitus, n (%)	25 (15)	11 (22)
Current smoker, baseline, n (%)	39 (24)	14 (28)
Smoking at 3 months follow-up, n (%)	21 (13)	7 (14)
Index STEMI, n (%)^b^	95 (58)	21 (42)
Anterior wall STEMI, n (%)	43 (26)	7 (14)
Troponin T max, ng/l (NSTEMI)^a^	204 (894)	161 (196)
Troponin T max ng/l (STEMI)^a^	3557 (4052)	2681 (7291)
Symptom to needle time (STEMI), hours ^a, b^	3.5 (3)	4.2 (6)
Number of stents implanted^c^	1.8 (1.1)	2.44 (1.3)
Management		
Participation in cardiac rehabilitation, n (%)	118 (73)	35 (70)
Beta-blocker at discharge, n (%)	124 (76)	38 (78)
Beta-blocker at 3 months, n (%)	118 (72)	33 (67)
ACEI at discharge, n (%)	71 (43)	17 (34)
ACEI at 3 months, n (%)	69 (42)	14 (28)
Intensive dose statin at discharge, n (%)	152 (93)	46 (92)
Intensive dose statin dose at 3 months, n (%)	145 (88)	43 (86)

Unless otherwise indicated continuous variables are presented as mean ± SD and categorical variables as absolute numbers and percentages

*bpm* beats per minute; *MI* myocardial infarction; *PCI* percutaneous coronary intervention; *CABG* coronary artery bypass grafting; *STEMI* ST-elevation MI; *NSTEMI* Non-ST-elevation MI; *ACEI* angiotensin converting enzyme inhibitors; *IQR* interquartile range

^a^Median and interquartile range

^b^p < 0.05

^c^p < 0.001. The dose of beta-blockers was expressed as equivalent to metoprolol succinate, and statins expressed as intensive (corresponding to atorvastatin ≥ 80 mg or rosuvastatin ≥ 20 mg od), or not

**Fig. 2:**
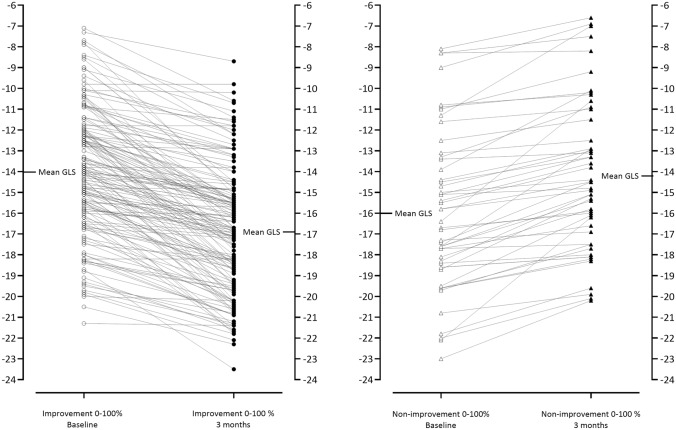
Individual GLS values at baseline and 3 months. Individual GLS values among patients with improvement (n = 164) to the left and with non-improvement (n = 50) to the right. Mean GLS at the two examinations are denoted as horizontal lines for both groups. *GLS* global longitudinal strain

**Fig. 3:**
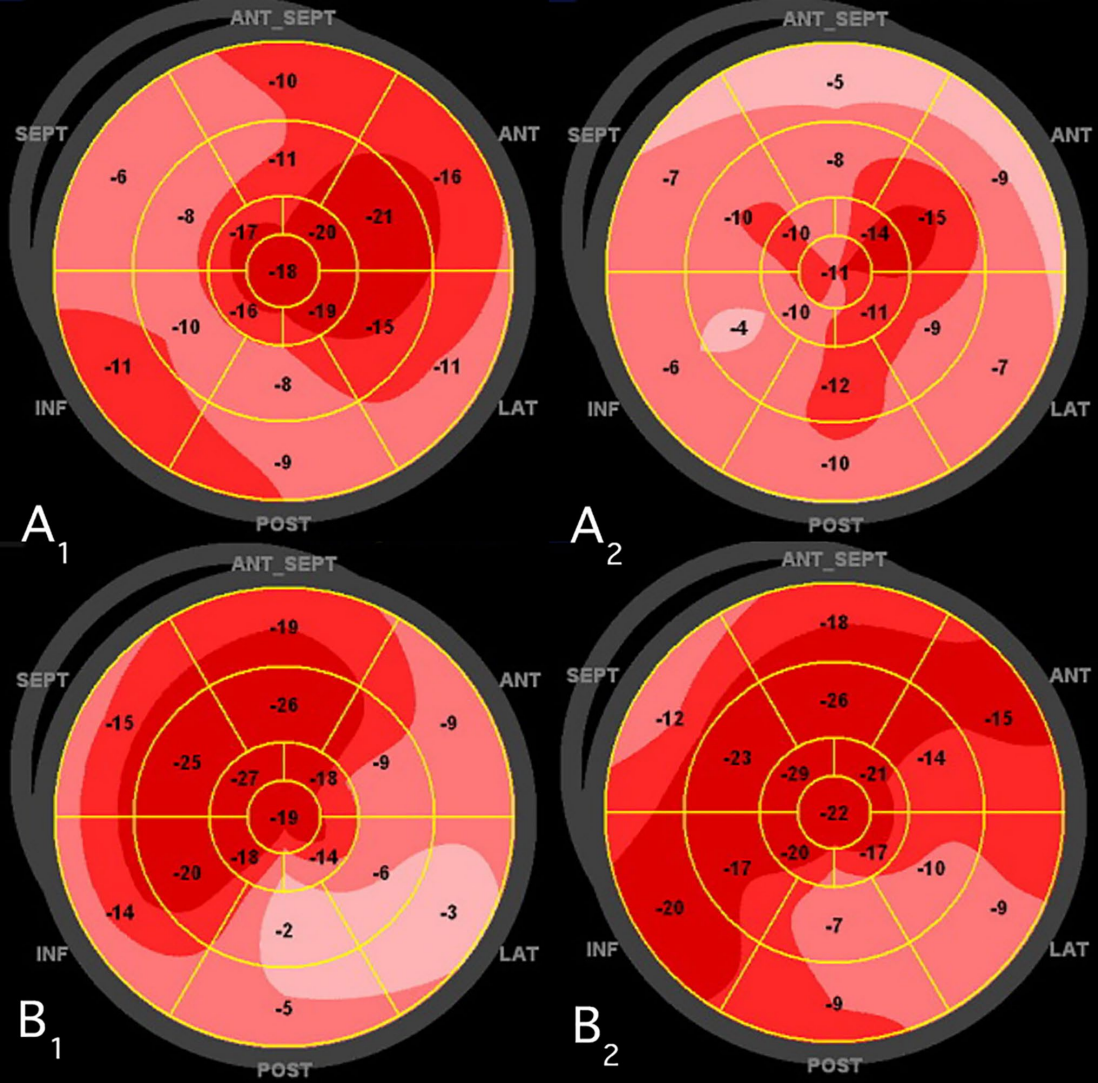
Illustration of a patient with non-improvement and a patient with improvement of GLS 3 months from baseline. **a** Bull’s eye plot from a patient with non-improvement of GLS from − 13.8% (A_1_) at baseline to − 10.1% (A_2_) after 3 months. Coronary angiography 7 days after symptom onset revealed native right coronary artery and a bypass to right posterior descending artery from previous coronary artery bypass surgery as culprit of a new non-ST-segment elevation myocardial infarction. Both lesions were treated with percutaneous coronary intervention. Maximum troponin T level was 116 ng/L. **b** Bull’s eye plot from a patient with improvement of GLS from − 14.9% (B_1_) at baseline to − 17.3% (B_2_) at 3 months. Coronary angiography 2 days after debut of symptoms found an occluded circumflex artery, which was treated with percutaneous coronary intervention. No other significant coronary artery stenosis were found. Maximum troponin T level was 3553 ng/L

Conventional echocardiographic data from the two groups are shown in Table 2, revealing no significant intergroup differences. Individual values for GLS at baseline and 3 months are presented in Fig. 2. The greatest improvement tended to occur among patients with the worst LV function. An opposite trend was noted among non-improvers. The correlation between baseline and delta GLS (change after 3 months) was strong (rho = 0.48, supplemental file 3). Bull’s eye plots from one patient with improvement and one with non-improvement at baseline and at 3 months follow-up are shown in Fig. 3.

**Table 2:** Conventional echocardiographic variables compared between improvers and non-improvers at baseline and 3 months

Variable	Baseline		3 months	
	Improvement	Non-improvement	Improvement	Non-improvement
LVEF, %	50 (7)	51 (9)	52 (7)	51 (9)
LVEDVI, ml/m^2^	84 (20)	81 (19)	85 (19)	82 (21)
LVESVI, ml/m^2^	41 (15)	40 (15)	41 (14)	41 (16)
Max LAVI, ml/m^2^	32 (9)	32 (10)	33 (11)	33 (11)
E/e’	10 (3)	10 (2)	10 (3)	11 (4)
LVMI, g/m^2^	134 (44)	129 (58)	128 (40)	132 (42)

Unless otherwise indicated, data are expressed as mean (± SD)

*GLS* global longitudinal strain; *LVEF* left ventricular ejection fraction; *LVEDVI* left ventricular end diastolic volume index; *LVESI* left ventricular end-systolic volume index; *Max* maximum; *LAVI* left atrial volume index; *LVMI* left ventricular mass index

**Fig. 4:**
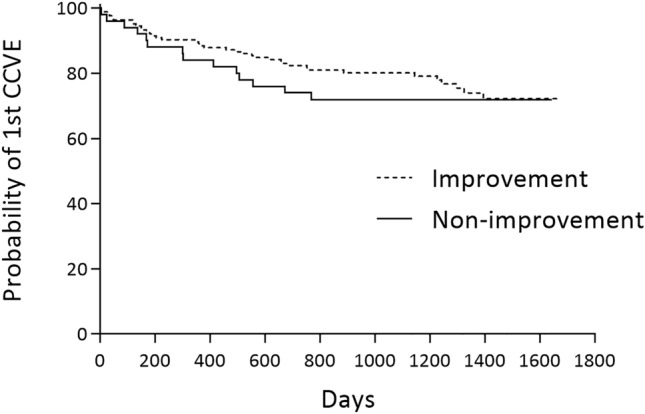
Kaplan–Meier curves reflecting time to first CCVE among patients with non-improvement and improvement. Kaplan–Meier curves reflecting time to first CCVE among patients with non-improvement and improvement. (Chi square 0.4 on 1 degrees of freedom, p = 0.5). *CCVE* composite cardiovascular events

During a mean follow-up period of 3.3 years (95% CI 3.2–3.4), 77 CCVE occurred in 52 patients (Table 3). Of the 23 angina events, all with angiographically verified progression, 18 were treated with urgent PCI and the remaining five had poor peripheries. The majority of recurrent AMI was NSTEMI (89%), and urgent revascularization was performed in 9/18 of these events (8 PCI and 1 CABG). All 12 AF cases were subjected to treatment with a novel anticoagulant and predominantly rate control. None had cardioversion at the time of new-onset AF diagnosis. A Kaplan–Meier plot reflecting time to first CCVE among patients with non-improvement and improvement are shown in Fig. 4.

**Table 3:** Number, categories and order of CCVE registered from first follow-up at 3 months to end-of follow-up

Endpoints	1st event	2nd event	3rd event	4 th event	Total
Death	4	1	2		7
Recurrent MI	11	3	3	1	18
Hosp., heart failure	4	3	1		8
Hosp., AP*	17	4	2		23
Hosp., VT	1				1
New-onset AF	8	4			12
Hosp., TIA/Stroke	7	1			8
Total, n	52	16	8	1	77

The columns refer the number of patients that had the specific endpoint registered as their first, second, third and fourth event during follow-up

*CCVE* composite cardiovascular events; *MI* myocardial infarction; *Hosp* Hospitalization; *AP* angina pectoris; *VT* ventricular tachycardia; *AF* atrial fibrillation; *TIA* transitoric ischemic attack

*AP with angiographic progression

*Model 1* of the Cox regression analysis is presented in Table 4. For *first* CCVE, using the age group 40–50 years as reference, only belonging to the 50–60 years decade (p < 0.05) was associated with lower incidence of CCVE. For *all* CCVE, patients in age decades 50–60 (p < 0.01) and 60–70 (p < 0.05) had a lower incidence than those in the age decade 40–50 years. In addition, baseline GLS but not GLS changes after 3 months was significantly associated with the number of CCVE (p < 0.05).

**Table 4:** Multi-adjusted hazard ratios for first and all CCVE during 3 years follow-up (Model 1)

	First CCVE episode		All CCVE episodes	
	Hazard ratio	95% CI	Hazard ratio	95% CI
Age category, years				
40–50	1	Reference	1	Reference
50–60	0.23*	0.07, 0.73	0.22**	0.08, 0.61
60–70	0.49	0.20, 1.18	0.46*	0.21, 1.00
70–80	0.69	0.28, 1.68	0.71	0.32, 1.54
80–90	1.37	0.45, 4.19	1.08	0.43, 2.69
Gender				
Women	1	Reference	1	Reference
Men	1.72	0.81, 3.65	1.36	0.72, 2.57
GLS baseline	1.09	0.99, 1.19	1.12**	1.03, 1.21
Δ GLS				
Non-Improvement (0–100%)	1	Reference		
Improvement (0–100%)	0.73	0.39,1.37	0.63	0.36, 1.10

*CCVE* composite cardiovascular events; *CI* confidence interval; *GLS* global longitudinal strain. Model 1 adjusted for age decades, gender, and GLS

*p < 0.05

**p < 0.001

In the more comprehensive *Model 2* (Table 5), belonging to the age decade 50–60 years was again associated with a lower incidence of *first* CCVE than those aged 40–50 years (p < 0.01). Previous AMI had a significant relationship with a higher incidence of *first* CCVE (p < 0.05). For *all* CCVE, the same pattern was found where patients in the decade 50–60 (p < 0.01), but also 60–70 years (p < 0.05) had a significantly lower incidence than those aged 40–50 years, and a history of previous AMI was a significant predictor of increased CCVE (p < 0.05). In this model neither baseline GLS nor GLS changes after 3 months were significantly associated with the incidence of subsequent CCVE.

**Table 5:** Multi-adjusted hazard ratios for first and all CCVE during 3 years follow-up (Model 2)

	First CCVE episode		All CCVE episodes	
	Hazard ratio	95% CI	Hazard ratio	95% CI
Age category, years				
40–50	1	Reference	1	Reference
50–60	0.20**	0.06, 0.66	0.19**	0.06, 0.60
60–70	0.41	0.16, 1.05	0.39*	0.18, 0.86
70–80	0.62	0.25, 1.57	0.61	0.27, 1,37
80–90	1.89	0.35, 3.61	0.74	0.27, 2.09
Gender				
Women	1	Reference	1	Reference
Men	1.62	0.76, 3.46	1.21	0.62, 2.37
GLS baseline	1.06	0.94, 1.19	1.07	0.96, 1.20
Δ GLS				
Non-improvement (0–100%)	1	Reference	1	Reference
Improvement (0–100%)	0.83	0.44, 1.57	0.66	0.37, 1.18
Previous MI	2.19*	1.12, 4.27	2.20*	1.14, 4.23
Anterior wall MI	1.09	0.55, 2.13	1.43	0.74, 2.75
EF, Baseline	1.01	0.95, 1.03	0.99	0.95, 1.93

*CCVE* composite cardiovascular events; *CI* confidence interval; *GLS* global longitudinal strain, *MI* myocardial infarction; *LVEF* left ventricular ejection fraction. Model 2 adjusted for age decades, gender, GLS, previous AMI, STEMI with anterior wall location and baseline LVEF

*p < 0.05

**p < 0.01

## Discussion

In the present study baseline GLS, but not GLS changes after 3 months, was significantly associated with the number of CCVE. To our knowledge, this is the first study to assess the prognostic importance of non-improved vs. improved GLS after 3 months in a post AMI population with the majority of patients having preserved LVEF.

No patients have been lost to follow-up after 3 months, and adequate hospital records have been available in this prospective, single-center study. In general, baseline differences in a comprehensive dataset of clinical and echocardiographic variables were small. Patients with non-improvement needed more stents and had a slightly lower LVEF at baseline than improvers. Although improvers with STEMI appeared to have a shorter symptom-to-needle time, the number of patients with delays above 10 h was similar in the two groups. The study has incorporated detailed information of patient management both at baseline and after 3 months. No major intergroup differences were found for medical treatment or participation cardiac rehabilitation. We chose to exclude a minority of patients due to cardiac events before the second examination since events could have had important influence on LV function at 3 months. This design, however, has a trend for selection of low-risk post-AMI patients. We believe that our findings are valid for the population studied, although obviously limited to the occurrence of coronary events and not for development of more serious endpoints such as heart failure or fatalities. Another matter to clarify is the possible influence of a regression to the mean phenomenon versus the influence of true biological changes of GLS after 3 months. Clearly, the changes cannot be explained by regression to the mean alone.

GLS improvement after 3 months can possibly be explained by myocardial stunning which is a reversible post-ischemic dysfunction of the myocardium that recovers during weeks to months [8, 15]. Patients with improvement had generally the worst LV function by GLS at baseline. Those with the poorest values may have had the highest potential for improvement. On the opposite, patients with non-improvement started with better LV function by GLS and turned out to have worse LV function than improvers at 3 months. Therefore, it may well be that the deterioration of LV function seen among patients with relatively good GLS values at baseline have resulted in a clinical course well comparable with improvers with poorer GLS values at baseline. The role of baseline GLS as predictor of CCVE was confirmed for all CCVE in the simple Model 1, but was not significant in the more comprehensive Model 2. Model 2 comprised several important confounders such as baseline LVEF, anterior wall STEMI and, especially, previous AMI, which may have influenced the prognostic role of baseline GLS, as opposed to the findings in most other studies. To us, this observation was surprising and may also be the result of a relatively small numbers of patients and events. On the other hand, most previous studies have evaluated the prognostic role of GLS on the occurrence of more serious endpoints such as fatalities and heart failure [4, 5], and not predominantly coronary events such has angina and recurrent AMI as in our study.

So far, studies exploring the association between echocardiographic assessment of changes in systolic function over time and outcome have mostly incorporated conventional echocardiographic measurements [7, 16]. The superior prognostic role of baseline GLS compared to LVEF has been verified in several larger studies incorporating the incidence of a broader selection of clinical endpoints such as higher numbers of mortality, ventricular arrhythmias, heart failure and reinfarctions [3, 7, 17–21]. Neither of these studies, however, had included GLS changes as a co-variable in adjusted analyses.

Only a few studies have followed the time course of GLS in AMI patients. Antoni et al. [8] arbitrarily defined improvement as ≥ 10% relative increase of baseline GLS and overall mean GLS improved by 17%, with 54% of their patients being categorized as improvers after 3 months. In the study of Baron et al. [9] a 10% relative GLS improvement was reported after 1 year, and improvement in GLS was associated 3 months with impairment of LV function (by LVEF, WMSI or GLS) at baseline. A Polish study by Wdoviak-Okrojec et al. [22] studied the improvement of regional systolic longitudinal strain on days 1,2,3,7, 30 and 180 days following AMI treated with PCI. The largest improvement occurred between days 1 and 2, with non-significant improvement thereafter. Neither of these studies reported the incidence of non-fatal cardiac events that might have influenced GLS changes during follow-up. In context of the findings in the Polish study [22], it is of importance that our baseline GLS has been derived at the time when the most pronounced improvement may have had taken place. In spite of this, we still observed an increase at a later time point, as also observed in the two aforementioned studies [8, 9].

An interesting finding was the non-linear association of CCVE with age. The youngest patients (i.e. 40–50 year) seemed to have a significantly higher risk of recurrent CCVE than the middle-aged (i.e. 50–60 and, in part 60–70 years). A possible explanation might be a more severe disease among those who sustain AMI at a younger age, or higher prevalence of unhealthy lifestyle behavior, as recently documented in a representative Norwegian post-AMI study [23]. Clearly, larger prospectively conducted long-term follow-up studies are needed to further explore the younger subgroup and eventually detect an “age paradox”.

One important question is whether a repeated echocardiographic examination including GLS measurements is necessary for prognostic purposes after 3 months or more. Apparently, from the results of our multi-adjusted analyses, this would seem unnecessary. On the other hand, in patients with poor baseline GLS the finding of major improvement at 3 months will render valuable prognostic importance for both the patient and treating physician. With our limited number of patients and events in mind, larger studies are needed before recommending such controls in a broader scale.

The major limitation of this study is the relatively small number of patients and a low number of events. In order to optimize the strength of our findings we included both first and subsequent events to represent all events. In addition, we added new onset AF among CCVE, which by many would be regarded as a minor event. This was, however, based upon our own experience from the ACTION study [24], where new-onset AF with the same diagnostic criteria as in the present study was a significant harbinger of death and heart failure among patients with stable CHD and LVEF ≥ 40%. Of note in this context is that 90% of our patients had LVEF ≥ 40% at baseline. This prognostic role of AF in patients with CHD was further corroborated in a recent review [25]. Since AF represented only 15% of all events, the inclusion of AF is not considered to have any major impact on our findings. Although an exploration of degrees of improvement and non-improvement associated with the incidence of CCVE would have been desirable, we deliberately avoided a subdivision in several subgroups. In this study, such a subdivision would be hampered by type 2 errors.

## Conclusion

Baseline GLS was significantly associated with the number of CCVE in revascularized AMI patients whereas non-improvement in GLS over 3 months follow-up was not. Although these findings do not support a routine practice of repeated GLS measurements, further large-scale studies are needed to answer this question.

## Supplementary Information

Below is the link to the electronic supplementary material.

Supplementary file1 (DOCX 14 kb)

Supplementary file2 (DOCX 12 kb)

Supplementary file3 (PDF 207 kb)

## Data Availability

Data are available upon request.
