# Supplementary material for: Prognostic impact of non-improvement of global longitudinal strain in patients with revascularized acute myocardial infarction
Source: Int J Cardiovasc Imaging. 2021 Jul 29;37(12):3477–87. doi: 10.1007/s10554-021-02349-2 (PMC8604850; doi:10.1007/s10554-021-02349-2)
Supplement: Supplementary file 1 — Supplementary file1 (DOCX 14 kb) [file 10554_2021_2349_MOESM1_ESM.docx]

**Supplemental file 1.** Basic and echocardiographic variables of all patients included compared with the final cohort where echocardiography both at baseline and after 3 months were obtained.

| **Characteristics** | **Included at baseline**  **n = 236** | **Repeated echocardiograms**  **n = 214** | **p-value** |
| --- | --- | --- | --- |
| Age, years, mean ± SD | 65 ± 10 | 65 ± 10 | 0.95 |
| Females, n (%) | 59 (25) | 54 (24) | 0.96 |
| **Medical history, n (%)** |  |  |  |
| Previous MI | 41 (17) | 37 (17) | 0.91 |
| Previous PCI | 41 (17) | 37 (17) | 0.91 |
| Previous CABG | 14 (6) | 13 (6) | 0.99 |
| Diabetes mellitus | 44 (19) | 38 (17) | 0.74 |
| Current smokers | 64 (27) | 59 (27) | 0.99 |
| Treated with β-blocker | 50 (21) | 47 (22) | 0.99 |
| Treated with ACEI | 89 (37) | 85 (38) | 0.88 |
| **MI subtype and PCI**  **procedure (median (IQR) unless otherwise specified** |  |  |  |
| STEMI, n (%) | 127 (54) | 116 (53) | 0.90 |
| Anterior wall STEMI, n (%) | 53 (23) | 49 (23) | 0.99 |
| STEMI TnT max, ng/L | 3393 (3964) | 3347 (3944) | 0.96 |
| NSTEMI TnT max, ng/L | 173 (463) | 18 (470) | 0.96 |
| STEMI, time to PCI in hours | 3.5 (3.0) | 4.0 (3.0) | 0.90 |
| NSTEMI, time to PCI in  hours | 48 (37) | 48 (37) | 0.83 |
| Number of arteries stented | 1 (1) | 1 (1) | 0.86 |
| Number of stents  implanted | 2 (2) | 2 (2) | 0.98 |
| Days from PCI to study  inclusion | 2 (1) | 2 (1) | 0.75 |
| **Echocardiographic variables, mean ± SD** |  |  |  |
| LVEF, % | 50 ± 8 | 50 ± 8 | 0.84 |
| LVEDVI, ml/m^2^ | 82 ± 20 | 82 ± 20 | 0.83 |
| LVESVI, ml/m^2^ | 42 ± 14 | 42 ± 15 | 0.82 |
| Max LA VI, ml/m^2^ | 32 ± 10 | 32 8± 10 | 0.81 |
| Max RV AI, ml/m^2^ | 12 ± 3 | 12 ± 3 | 0.90 |
| RV AF, % | 47 8± 9 | 47 ± 9 | 0.90 |
| E/E’ | 10.3 ± 3.2 | 10.3 ± 3.3 | 0.98 |
| LVMI, g/m^2^ | 133 ± 46 | 132 ± 45 | 0.90 |
| Diastolic dysfunction, n (%) | 8 (3) | 7 (3) | 0.94 |
| GLS, % | -14.4 ± 3.4 | -14.4 ± 3.3 | 0.93 |

Abbreviations: MI = myocardial infarction, PCI = percutaneous coronary intervention, CABG = coronary artery bypass grafting, TnT = Troponin T, STEMI = ST-elevation myocardial infarction, NSTEMI = non-ST-elevation Abbreviations: MI = myocardial infarction, PCI = percutaneous coronary intervention; CABG = coronary artery bypass grafting; ACEI = angiotensin converting enzyme inhibitors; IQR = interquartile range; STEMI = ST-elevation MI; TnT = troponin T; NSTEMI = Non-STE elevation MI; ECHO = echocardiography; LVEF = left ventricular ejection fraction;, LVED = LV end-diastolic, LVESVI = LV end-systolic; VI = Volume index. Max LA VI = maximum left atrial VI; Max RV AI = maximum right ventricular area index; RV AF = RV area fraction, E/E’ = ratio of E to E’, LVMI = LV mass index. GLS = global longitudinal strain.
