# Supplementary material for: Prognostic impact of non-improvement of global longitudinal strain in patients with revascularized acute myocardial infarction
Source: Int J Cardiovasc Imaging. 2021 Jul 29;37(12):3477–87. doi: 10.1007/s10554-021-02349-2 (PMC8604850; doi:10.1007/s10554-021-02349-2)
Supplement: Supplementary file 2 — Supplementary file2 (DOCX 12 kb) [file 10554_2021_2349_MOESM2_ESM.docx]

**Supplemental file 2:** Echocardiographic variables at baseline and three months, with percentual change from baseline (n=214)

| **Variable** | **Baseline** | **3 months follow-up** | **Change from baseline (%)** | **p-value** |
| --- | --- | --- | --- | --- |
| GLS, % | -14.4 (3.3) | -16.2 (3.4) | 12.5 | <0.001 |
| LVEF, % | 50 (8) | 52 (7) | 4.0 | <0.001 |
| LVEDVI, ml/m^2^ | 83 (20) | 84 (20) | 1.2 | 0.07 |
| LVESVI, ml/m^2^ | 42 (15) | 41 (15) | 2.4 | 0.03 |
| Max LAVI, ml/m^2^ | 31 (10) | 34 (11) | 6.2 | 0.03 |
| E/e’ | 10.2 (3.2) | 9.9 (3.3 | 2.9 | 0.13 |
| LVMI, g/m^2^ | 133 (47) | 130 (41) | 2.3 | 0.23 |
| Heart rate, bpm | 70 (12) | 67 (11) | 4.3 | <0.001 |
| SBP, mmHg | 127 (18) | 129 (18) | 1.6 | 0.047 |

Abbreviations: GLS: Global longitudinal strain; LVEF: Left ventricular ejection fraction; LVEDVI: Left ventricular end diastolic volume index: LVESVI: Left ventricular end-systolic volume index; Max: maximum; LA: Left atrial; LVMI: Left ventricular mass index; bpm: beats per minute; SBP: Systolic blood pressure.
