# Supplementary material for: Prognostic impact of non-improvement of global longitudinal strain in patients with revascularized acute myocardial infarction
Source: Int J Cardiovasc Imaging. 2021 Jul 29;37(12):3477–87. doi: 10.1007/s10554-021-02349-2 (PMC8604850; doi:10.1007/s10554-021-02349-2)

Supplemental file 3: Scatterplot showing the correlation between baseline GLS% (abscissa) and delta (percentual change of the baseline value after three months) GLS (ordinate). rho = 0.48 .

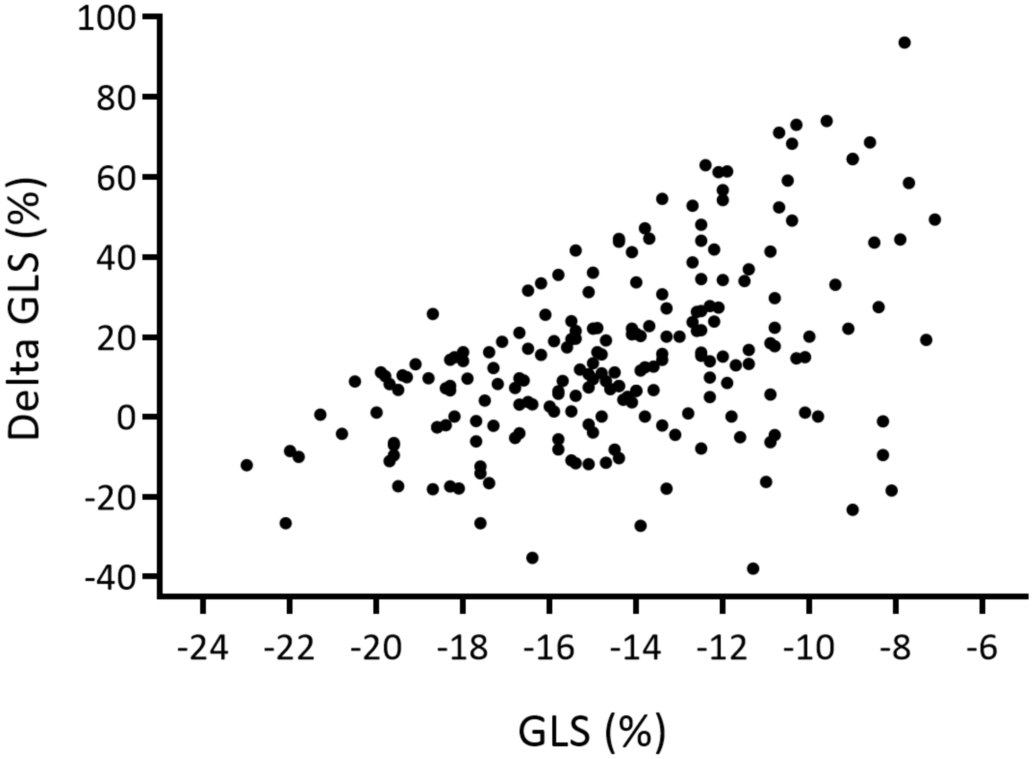

Supplement: Supplementary file 3 — Supplementary file3 (PDF 207 kb) [file 10554_2021_2349_MOESM3_ESM.pdf]
